# Nature’s Gold (Moringa Oleifera): Miracle Properties

**DOI:** 10.7759/cureus.26640

**Published:** 2022-07-07

**Authors:** Sonali B Rode, Amruta Dadmal, Harsh V Salankar

**Affiliations:** 1 Department of Pharmacology, Jawaharlal Nehru Medical College, Datta Meghe Institute of Medical Sciences, Wardha, IND; 2 Department of Clinical Research, School of Allied Health Sciences, Datta Meghe Institute of Medical Sciences, Wardha, IND; 3 Department of Pharmacology, Datta Meghe Medical College, Datta Meghe Institute of Medical Sciences, Wardha, IND

**Keywords:** flavonoids, anti-microbial, anti-oxidant, anti-inflammatory, medicinal properties, moringa oleifera

## Abstract

*Moringa oleifera*, known as a miracle tree, is a small plant cultivated all over the world due to its multiple medicinal uses.It is cultivated for its nutritious pods, edible leaves, and flowers which are very helpful as food, medicine, cosmetic oil, and forage for livestock. It is a good source of protein, oils, vitamins, fatty acids, micro-macro mineral elements, and various phenolics. Its roots, bark, gum, leaf, fruit (pods), flowers, seed, and seed oil possess various biological activities. The main flavonoids found in its leaves are myricetin, quercetin, and kaempferol.

Each part of the *Moringa oleifera* tree is used for a variety of nutritional and medicinal purposes. The tree has anti-inflammatory, antimicrobial, antioxidant, anticancer, antihypertensive, hepatoprotective, anti-ulcer, antifertility, and diuretic properties. Its many pharmacological benefits are exploited as therapeutic remedies in the traditional medicinal system for various diseases.

Moringa’s hypolipidemic, antihypertensive, antioxidant, anti-cancer, anti-diabetic, and hepatoprotective properties have been attributed to quercetin, phenolic acid, tannins, and saponins. More research into this remarkable healer could lead to the creation of new drugs to treat a variety of illnesses. This article gives a quick summary of the medical potential of Moringa and its future as a component of modern medicine. According to the findings of this study, Moringa needs to be properly evaluated before it can be used as a medication in modern medicine.

## Introduction and background

*Moringa oleifera* (*M. oleifera*) is the widely cultivated species of the family Moringaceae, native to the Indian subcontinent [1]. Its common names are Moringa, Drumstick tree (due to the long, slender, triangular seed-pods), Horseradish tree (due to the taste of the roots that resemble horseradish), and Ben oil tree or Benzolive tree [2]. M. oleifera has a maximum height of 10-12m (32-40 ft) and a trunk diameter of 45cm (1.5ft). The fruit is a brown capsule with three sides with a size of 20-45cm, which holds a globular, dark brown seed diameter of about 1cm. The seeds have three whitish papery wings, which are dispersed by wind and water [3].

The Moringa tree is mainly adapted to tropical and subtropics. It thrives best in direct sunlight under 500 meters altitude. It may grow in a variety of soil conditions but favors a well-drained sandy and loamy soil that is neutral to a slightly acidic pH (6.3-7.0) [4]. The tree requires an average daily temperature of 25-30 degrees Celsius for optimum leaf and pod production, although the tree can tolerate up to 48 degrees Celsius in the shade and can survive a light forest [5]. Moringa seeds are planted as soon as they mature because they have no dormancy period, and they retain the ability to germinate for a year [4]. The scientific classification of Moringa is shown in Table 1.

**Table 1:** Scientific classification of Moringa oleifera

1	Kingdom	Plantae
2	Order	Brassicales
3	Family	Moringaceae
4	Genus	Moringa
5	Species	M. Oleifera

## Review

Nutritional facts

1. Fresh leaves from *M. oleifera* are a good source of vitamin A. It is well established that vitamin A has important functions in vision, reproduction, embryonic growth and development, immune competence, and cell differentiation [6,7].

2. Vitamin C is one of the most abundant nutrients in Moringa. A single serving of Moringa contains more Vitamin C than an orange by weight [8].

3. 100 grams of raw moringa leaves provide 4 milligrams of iron, which are corresponding to about 22% of the average recommended dietary allowance. That's much higher than spinach, which has only about 2.7 mg of iron per 100 grams of raw spinach. Moringa powder has about 0.5 mg of iron per teaspoon, making it one with the highest nutritional value known in practically any plant [8].

4. Calcium is present in abundance in Moringa leaves [8].

5. Moringa has been shown to aid in depression, anxiety, and exhaustion [8].

6. Moringa seed oil protects hair and keeps it clean and healthy. Moringa also includes protein, which aids in the protection of skin cells against injury [8].

7. Moringa contains various amino acids, as depicted in Table 2.

**Table 2:** Various amino acids of Moringa and their role as nutrients

Sr. No.	Amino Acids	Function
1	Threonine	Required for the metabolism and it prevents accumulation in the liver
2	Leucine	Helps to boost energy levels
3	Isoleucine	Excellent for the brain
4	Phenylalanine	Required for the communication between the nerve cells of the brain, improving memory and developing alertness
5	Tryptophan	Required to support the immune system, and boost mood. It helps to lower LDL cholesterol levels
6	Lysine	Helps in the retention of calcium in the bones, it also holds up antibodies and impedes the growth of virus cells

Bioactive components

The high number of bioactive compounds might explain the pharmacological properties of *M. oleifera* leaves. Leaves have been used for the treatment of various diseases, from malaria and typhoid fever to hypertension and diabetes.

*M. oleifera* is a rich source of flavonoids that gives protection to various chronic diseases, including cardiovascular diseases, diabetes, and cancer. Flavonoids, present in the plant, have a benzo-γ-pyrone ring as a common structure. The main flavonoids found in *M. oleifera* leaves are myricetin, quercetin, and kaempferol [7].

Quercetin is found in dried *M. oleifera* leaves and seeds, which is a powerful antioxidant with many therapeutic benefits. Moringa’s hypolipidemic, antihypertensive, antioxidant, and anti-diabetic properties are attributed to quercetin. It can protect insulin-producing pancreatic β cells from Streptozotocin (STZ) induced oxidative stress and apoptosis in rats [7].

Phenolic acids are another bioactive compound, naturally present in plants, having antioxidant, anti-inflammatory, anti-diabetic, antimutagenic, and anticancer properties. Chlorogenic acid (CGA) is the main phenolic acid found in *M. oleifera*. CGA has anti-hypolipidemic properties, as it reduces plasma total cholesterol and triglycerides (TG) in obese Zucker rats or mice fed a high-fat diet and reverses STZ-induced dyslipidemia in diabetic rats [7].

Tannins are water-soluble phenolic compounds that precipitate alkaloids, gelatin, and other proteins. It has been reported to have anticancer, antiatherosclerosis, antiinflammatory, and antihepatotoxic properties [7]. Alkaloid Moringine relaxes bronchioles and has antiasthma properties [9]. Saponins present in the leaves have anti-cancer properties [7].

Medicinal properties

Every part of the Moringa tree is utilized for a variety of nutritional and therapeutic purposes, which has been aptly demonstrated in various animal studies [10].

Anti-inflammatory activity

The anti-inflammatory effects of aqueous and ethanolic (90 percent) extracts of leaves of *M. oleifera* Lam have been investigated in male albino rats. Both extracts showed maximum action within two hours of the challenge. In the first hour after carrageenan injection, the aqueous extract caused considerable edema suppression, similar to that seen with Ibuprofen. The results confirmed the anti-inflammatory effect of the plant [11]. The edema suppression action exhibited by the drug may be due to the inhibitory effects on the release of histamine, 5-hydroxy tryptamine, and kinin-like substances, which are reported to release from mast cell degradation during the first hour of carrageenan-induced artificial paw edema.

The anti-inflammatory effect of an aqueous decoction of the root has been studied in rats with weights of 120 and 160 gm, where edema was induced in the rat paw subcutaneously by injection of carrageenin. The rat-paw volume was calculated one, three, and five hours after injection of carrageenin and was compared at the same intervals after treatment with *M. oleifera* decoction. The results were compared with the anti-inflammatory effects shown by indomethacin in the same study model [12].

Anti-oxidant property

Extracts of *M. oleifera* leaf were examined in two stages of maturity to study the mechanism of pharmacological actions and antioxidant activity in the standard in-vitro model. Consecutive aqueous leave decoction of *M. oleifera* exhibited effective scavenging upshot free radical of 2, 2-diphenyl-2-picryl hydroxyl (DPPH), nitric oxide radical, superoxide, and inhibition of lipid peroxidation. This prevents oxidative damage to major biomolecules and affords significant protection against oxidative damage [13].

The antioxidant activity of *M. oleifera* has been also studied where the leaves of the tree were excluded with methanol, dichloromethane, ethyl acetate, and n-hexane. The methanol decoction showed the highest free radical scavenging action with IC50 in the 1,1-diphenyl-2-picrylhydrazyl assay and ABTS assay [14].

Anti-microbial property

The extract of *M. oleifera* leaves, roots, and seeds have shown antimicrobial properties. The leaf decoction was tested for antimicrobial action against different microorganisms like gram-negative- *Escherichia coli*, *Staphylococcus aureus*, *Sarcina lutea*, gram-positive- *Bacillus cereus*, *Bacillus subtilis,* and acid-fast *Mycobacterium phlei* using disc diffusion assay method measured in terms of zone of inhibition. According to the result, it was revealed that the decoction had bactericidal effects on all the organisms. [15].

Anti-asthmatic activity

The efficacy and safety of *M. oleifera* dry seed kernels were studied in 25 mild-to-moderate asthma patients for three weeks. A spirometer was used to evaluate clinical efficacy in terms of symptoms and respiratory functioning before and after the treatment. Marked advancement was observed in forced vital capacity, forced expiratory volume in one second, and peak expiratory flow rate in asthmatic patients. None of the patients showed any adverse effects. Moringine, an alkaloid present in Moringa relaxes bronchioles. It resembles ephedrine in action and is useful in the treatment of asthma [9].

Anti-diabetic activity

Animal studies had suggested that Moringa helps to lower blood sugar levels. Antidiabetic action of its seed on streptozotocin (STZ) induced diabetic male rats was examined, where immunoglobulins (IgA, IgG), fasting blood sugar, and glycosylated hemoglobin levels were improved, and the normal histology of the kidney and pancreas were restored in moringa treated groups, compared to the diabetic positive control group [16]. The use of *M. oleifera* in diabetic patients improved glucose tolerance by decreasing post-prandial sugar levels after one, two, and three months of treatment [17].

Analgesic effect

Pain is an unpleasant sensation that is often the only symptom used to diagnose a variety of disorders. Analgesic medications without side effects are in high demand. The leaves of *M. oleifera* are a possible source of phytochemical compounds with analgesic properties.

A randomized controlled study used the acetic acid-induced writhing test and eddy's hot plate test to assess *M. oleifera's* analgesic effectiveness. In each of the two experimental models, a total of 36 mice were used. Group I: Control (normal saline); Group II: Standard (diclofenac); Group III, IV, V, VI (ethanolic extract of *M. oleifera*). In all models, Moringa leaves showed analgesic activity, indicating that it has both central and peripheral analgesic effects [18].

Anti-pyretic activity

Fever and inflammation, symptoms of a variety of illnesses, can be treated with modern medications, although they have few adverse effects. Several investigations are being conducted around the world to find natural antipyretic drugs that are more effective and have fewer or no side effects. *M. oleifera* bark had shown antipyretic properties against *E. coli-*induced pyrexia to have antipyretic efficacy in rabbits, supporting its ethnopharmacological use as an antipyretic herb [19].

Anti-hypertensive activity

Effects of *M. oleifera* leaves extract were studied on isolated frog hearts for antihypertensive action. Alkaloidal salts present in Moringa showed a negative inotropic effect on the frog heart and guinea pig ileum. Isothiocyanate and niaziminin, present in Moringa are responsible for antihypertensive action [20].

Diuretic activity

The diuretic efficacy of an alcoholic extract of *M. oleifera* leaves was evaluated in six groups of six Swiss albino rats each. The first group (Control) was given 25mg/kg of normal saline. The second group (Standard) was given 25 mg/kg of hydrochlorothiazide. Third (Test I), fourth (Test II), and fifth group (Test III) were administered *M. oleifera* at 50 mg/kg, 100 mg/kg, and 200 mg/kg, respectively. Urine collection was done after five hours and 24 hours using a metabolic cage. *M. oleifera* leaf extract had a dose-dependent diuretic effect that was higher than the control but lower than hydrochlorothiazide. Urinary volume excretion of test groups I, II, and III was significant when compared with control but lower in comparison with a standard. Sodium excretion was significant with 50 and 100 mg of the test drug but decreased with 200 mg of the test drug. Chloride excretion was increased with all three test groups, whereas potassium excretion was increased only with 100mg and 200 mg doses. Potassium-sparing activity, on the other hand, was not found [21].

Cholesterol-lowering property

Moringa not only lowers the production of cholesterol in the body but also stops its accumulation in the veins, lowering the risk of heart attacks and strokes. A study was done to find the optimal combination of cholesterol-lowering potential of a blend of methanol extracts of *M. oleifera* leaf and fruit and to test the extracts in hypercholesterolemic animal models. The extract mix exhibited a significant reduction in serum triglyceride levels after three and six weeks of therapy in a high cholesterol diet animal. The findings suggested that the *M. oleifera* blend may lower cholesterol and triglyceride levels by limiting cholesterol absorption and that it might be developed as a standardized blend for the dietary supplement market [22].

Anti-tumor activity

Moringa extracts have characteristics that may aid in the prevention of cancer. It also contains niazimicin, a chemical that inhibits the growth of cancerous cells [8]. The anticancer effect of the *M. oleifera* flower trypsin inhibitor (MoFTI) was tested in sarcoma 180-bearing mice in this study. In comparison to the control group, treated animal tumors had fewer secondary vessels and smaller primary vessels. Water and food consumption, as well as body and organ weights, showed no significant changes. The liver, kidneys, and spleen were not damaged, according to the histopathological investigation. Results demonstrated anticancer activity while exhibiting no toxicity [23].

Hepatoprotective activity

Moringa appears to protect the liver from anti-tubercular medication damage and can speed up the healing process [9]. In rats, the hepatoprotective efficacy of an ethanolic extract of *M. oleifera* leaves was tested against damage to the liver caused by antitubercular medicines such as Isoniazid, Rifampicin, and Pyrazinamide. The effect of oral administration of the extract on serum levels of liver enzymes revealed a significant protective effect. Histopathological study of liver sections corroborated this observation. The findings of this investigation revealed that therapy with *M. oleifera* extracts seemed to improve recovery from antitubercular drug-induced liver damage [24].

Anti-fertility effect

The estrogenic, antiestrogenic, progestational, and antiprogestational properties of an aqueous extract of *M. oleifera* roots were examined in rats. Uterine weight of bilaterally ovariectomized rats increased with oral treatment along with stimulation of uterine histological structure showing estrogenic action. When the extract was administered in combination with estradiol dipropionate (EDP), the uterus wet weight was reduced compared to when EDP was given alone, and uterine histological structure was suppressed. In the deciduoma test, the highest dose of Moringa inhibited the formation of deciduoma in 50% of the rats, indicating its antiprogestational action [25].

Anti-ulcer potential

An ethanolic root-bark extract of *M. oleifera* has been tested for its antiulcer property in albino Wistar rats with the help of two experimental models: ethanol-induced and pylorus ligation-induced gastric ulceration. For 15 days, the extract was supplied orally at three various doses. The antiulcer activity in rats was determined and compared statistically with the antiulcer effects in the control rats treated with saline (NaCl). The *M. oleifera* reduced the ulcer index approximately as compared to the control group (omeprazole). This study suggested that *M. oleifera* acquires valuable antiulcer and antisecretory and is used as a source for an antiulcer medication [26].

## Conclusions

Even though this article presented glimpses of *M. oleifera's* uses for performing an appraisal of this promising nutrition and medicinal plant, the therapeutic potential of *M. oleifera* is extensive and difficult to address in a single article. Although many bioactive chemicals have been found in Moringa, our understanding of its complete reserve is still in its infancy. Future rigorous investigations aimed at detecting and commercializing *M. oleifera* bioactive chemicals could potentially lead to the development of treatments for a variety of illnesses. As a result, it can also demonstrate the authenticity of *M. oleifera*'s traditional use in diverse folklores.
